# Assessing equity and quality indicators for older people – Adaptation and validation of the Assessing Care of Vulnerable Elders (ACOVE) checklist for the Portuguese care context

**DOI:** 10.1186/s12877-022-03104-5

**Published:** 2022-07-06

**Authors:** Adriana Taveira, Ana Paula Macedo, Nazaré Rego, José Crispim

**Affiliations:** 1 Escola de Economia e Gestão, Universidade do Minho, Campus de Gualtar, 4710-057 Braga, Portugal; 2 Health Sciences Research Unit: Nursing (UICISA: E), Nursing School of Coimbra (ESEnfC), Coimbra, Portugal; 3 School of Nursing, University of Minho, Campus de Gualtar, 4710-057 Braga, Portugal; 4 NIPE, Escola de Economia e Gestão, Universidade do Minho, Campus de Gualtar, 4710-057 Braga, Portugal

**Keywords:** ACOVE-3, Cross-cultural adaptation, Healthcare access equity, Healthcare quality, Instrument validity, Portuguese, Vulnerable older people

## Abstract

**Background:**

Development has promoted longer and healthier lives, but the rise in the proportion of older adults poses new challenges to health systems. Susceptibilities of older persons resulting from lower knowledge about services availability, health illiteracy, lower income, higher mental decline, or physical limitations need to be identified and monitored to assure the equity and quality of health care. The aim of this study was to develop equity indicators for the Assessing Care of Vulnerable Elders (ACOVE)-3 checklist and perform the first cross-cultural adaptation and validation of this checklist into Portuguese.

**Methods:**

A scoping literature review of determinants or indicators of health (in)equity in the care of older people was performed. A total of 5 language experts and 18 health professionals were involved in the development and validation of the equity and quality indicators through expert opinion and focus groups. Data collected from focus groups was analyzed through directed or conventional content analysis. The usefulness of the indicators was assessed by analyzing the clinical records of 30 patients.

**Results:**

The literature review revealed that there was a worldwide gap concerning equity indicators for older people primary health care. A structured and complete checklist composed of equity and quality indicators was obtained, validated and assessed. A significant number of non-screened quality or equity related potential occurrences that could have been avoided if the proposed indicators were implemented were detected. The percentage of non-registered indicators was 76.6% for quality and 96.7% for equity.

**Conclusions:**

Applying the proposed checklist will contribute to improve the monitoring of the clinical situation of vulnerable older people and the planning of medical and social actions directed at this group.

**Supplementary Information:**

The online version contains supplementary material available at 10.1186/s12877-022-03104-5.

## Background

Further gains in average population health require that health equity is improved [1, 2]. The quality of care provided in an equitable health care system is independent of personal characteristics such as gender, ethnicity, geographic location or socio-economic status [3]. Health care equity entails equal *access to* and *utilization of* available care for equal need, and equal *quality of care* for all [4]. Since health inequalities are potentially avoidable [5], they should be a priority for policy makers and health managers.

A vulnerable older person (i.e., an older adult who is at risk of functional decline or death in a period of two years [6]) may be exposed to situations of inequity due to ageism [6, 7], social isolation and loneliness [8], higher mental decline, or physical limitations. Frequently, older adults accumulate other conditions that may be factors of inequity (such as, lower education levels, lower health literacy, and lower income) or increase the complexity of care (namely, multimorbidity and the associated polymedication, and geriatric syndromes). As the population structure changes and, especially in developed countries, the proportion of adults aged 65 or over rises [9, 10], the equity and quality of health services provided to vulnerable older adults should be monitored and assured. In a primary health care (PHC) standard consultation, managing the care of vulnerable older adults can be challenging, given their care needs and the limited time for each visit. Health care workers are often unprepared to effectively manage the health care needs of older adults; thus, concerted and sustained efforts of academic leaders and health professional groups directed at improving education and training are needed [11]. The availability of equity and quality indicators (EQI) focused on vulnerable older people that can guide health professionals is of special importance in this context.

The existence of quality indicators (QI) is an essential prerequisite to monitor, compare and improve the level of effectiveness and efficiency of health systems [12]. Several studies have addressed QI for specific issues related with vulnerable older persons: falls and mobility problems [13], hearing loss [14], pain management [15], medication [16], and undernutrition [17]. QI are already well established in national health systems [12, 18], but those related to vulnerable older adults are relatively new and require more time to be widely adopted. E.g., in Portugal, the 365 existing quality indicators for PHC [19] only include four indicators concerning the quality of health care for older adults. In 2000, RAND Health Care researchers released the first set of quality measures specifically developed for vulnerable older persons, the Assessing Care of Vulnerable Elders (ACOVE) project [20]. The initial ACOVE quality indicators covered 22 specific conditions of continuity and coordination of care for older people and were developed based on the literature and its authors’ expertise [21]. In ACOVE-3 [22], more clinical conditions were included. Several studies [23] have highlighted ACOVE [20] for its broad set of quality indicators. Its structure comprises an extensive variety of clinical conditions, suitable to measure the quality of care provided to vulnerable older adults [12, 24]. This instrument has been recognized by the scientific and health communities (namely, by WHO) for its reliability in the evaluation and definition of practical interventions.

On the other hand, monitoring of equity in health care is in its infancy and remains isolated from mainstream quality assurance [25]. In a literature review, Burkett et al. [26] compared quality indicators for older people care in emergency departments and concluded that ACOVE-3 [27] was one of the few quality instruments that comprehensively considered five of the six Health Care Quality dimensions proposed by the American Institute of Medicine [3]: aiming at *safe*, *effective*, *patient-centered*, *timely*, *efficient* health care. The sixth – *equitable* health care – had not been considered in any quality instrument [26]. To our best knowledge, until this study, this lack had not been fulfilled.

### Aims

The objectives of the research were: a) to develop a set of equity indicators (EI) for ACOVE-3 to be used by PHC providers, contributing to the improvement of health care to vulnerable older adults; b) to undertake the first cross-cultural adaptation and validation of ACOVE-3 into Portuguese; c) to assess the validity of the developed EQI and if they would be well accepted by health professionals. The study focused in PHC, the most cost-effective way of providing accessible care to chronic conditions and multimorbidity [28, 29].

## Methods

### Research design

The research followed a sequential research design that included: Phase 1) a literature review to understand the state-of-the art concerning the development of equity indicators for PHC of vulnerable older persons; Phase 2) a cross-cultural adaptation of ACOVE-3 to Portuguese, and Phase 3) the assessment of the validity and acceptance of the proposed EQI using expert opinions and focus groups. Table 1 presents an overview of the activities performed to develop the EQI checklist (detailed in the next subsections), and Additional file 1 describes the experts and focus groups participants, and the research tasks they were involved in.

**Table 1:** Phases of the equity and quality indicators checklist development

**Phase #1—Identification of equity indicators**
**Inputs**: Literature review protocol
**Activities**: Review the literature and choose equity indicators to be applied
**Outputs**: List of equity domains; list of selected equity standard descriptors for primary health care of older adults organized into domains
**Phase #2—Cross-cultural adaptation of ACOVE-3 and EI incorporation**
**Inputs**: ACOVE-3 checklist, list of equity domains, and list of selected equity standard descriptors for primary health care of older adults organized into domains to be included into ACOVE structure
**Activities** a) Discuss equity concept and the dimensions to be considered to identify situations of inequity—Focus Group 1; b) Discuss the best equity standard descriptors to be used—Focus Group 2; c) Perform a cross-cultural adaptation following the steps recommended by Beaton et al. [30]: 1) translation—Participant P1, 2) back-translation—Participant P5, 3) review of back-translation by independent reviewers—Participants P6, and P7, and 4) harmonization—Participants P2, P3, and P4; d) Insert the equity indicators into ACOVE structure (organized into clinical conditions)
**Outputs**: Complete list of equity and quality indicators (EQI); final list of operational definitions of the five equity dimensions (equity standard descriptors)
**Phase #3**—**EQI assessment**
**Inputs**: Complete list of EQI organized into clinical conditions;
**Activities**
a) Equity and quality indicators (EQI) face and content validation using expert opinions—Participants P2, P3 and P4; b) Discuss EQI face, content and responsiveness validity—Focus Groups 3 and 4; c) Discuss EQI acceptance—Focus Groups 3 and 4; d) Explore EQI usefulness—Past clinical records analysis
**Outputs**: Final list of EQI, and an overall appraisal of the benefits and acceptance of the checklist to propose

### Literature review

A scoping review of the literature published from 2016 to 2020 following PRISMA [31] and JBI’s Population (or participants), Concept, Context (PCC) [32] guidelines aimed at answering the question *What equity indicators are available for health care of older adults?* was performed in order to identify indicators suited for primary health care settings. The concept of interest was equity indicators or determinants, and the context was health care of older adults [32]. (Studies that did not concern PHC were eligible because excluding them would be too limitative.) Then, the inclusion criteria were: focusing on health equity; in the care older people; and identifying determinants or indicators of health (in)equity. Only original research was considered.

PubMed, Medline, and Web of Science were searched for ("healthcare" OR “health care” OR "primary health care") AND ("older people" OR "older person*" OR "older adult*" OR "elder*") AND "equity" AND "indicators" in titles or abstracts. Only documents written in English were considered. After duplicates were removed, the abstracts of the remaining studies were read and their full texts were skimmed in order to select a final set of studies that were fully and carefully read.

The purpose of the scoping review was merely descriptive and the analysis consisted on registering, for each article in the dataset, the equity dimensions and the equity determinants or indicators identified and if they were related to PHC or older adults in data extraction table.

### Cross-cultural adaptation

A cross-cultural adaptation of an assessment instrument involves the development of versions that are equivalent to the original but simultaneously linguistically and culturally adapted to the new specific context. ACOVE-3 instrument has twenty-six clinical conditions, but only twelve (identified as conditions of vulnerability of older adults) were considered in the adapted and translated instrument, since they: a) were not included in the National Health Program for the older people already implemented in the Portugal [33]; and b) can be used in PHC [33]. The clinical conditions considered were: continuity and coordination of care, dementia, depression, falls and mobility problems, hearing loss, medication use, pressure ulcers, screening and prevention, sleep disorders, malnutrition, urinary incontinence, and vision.

The ACOVE-3 checklist was translated into Portuguese following the Guidelines for Cross-Cultural Adaptation Process of Beaton et al. [30], a widely recognized and validated iterative method (Table 1). This process requires a review by an expert committee (that assures semantic, idiomatic, experiential, and conceptual equivalences). In this study, the committee was composed by six experts (Additional file 1): a) one specialized in the translation of data collection instruments in the clinical area, native in Portuguese (P1) that translated the English version into Portuguese; b) three experts in health care (P2, P3, P4); and c) two experts in applied language (P5, P6) that independently performed the backward translation (in a first moment, blinded to the original English version). The EI (in Portuguese) were incorporated into the ACOVE-3 checklist after expert P1 translated it from English to Portuguese. The experts were invited to make comments about their understanding of each item and its relevance, both on a personal and a cultural level, and made suggestions about items or terms they considered difficult to understand, offensive, redundant, or inappropriate.

The health care experts (P2, P3 and P4) assessed each of the 170 EQI (139 ACOVE-3 QI and 31 EI) by scoring it in terms of language understandability, syntax, and semantics using the 4-point Likert scale: 1—Unclear, 2—Slightly unclear, 3—Quite clear, 4—Extremely clear; and in terms of content relevance, using the scale: 1 – Not relevant, 2—Slightly relevant, 3—Quite relevant, 4—Extremely relevant. These scales are similar to those proposed by Hambleton et al. [34] and frequently used in healthcare. The index of content validity (CVI), the most widely reported measure of content validity, specifically the Average Scale-level-CVI (S-CVI/Ave) and the Universal Agreement Scale-level-CVI (S-CVI/UA) [35], were calculated using the responses. Face and content validity [36] were confirmed at this stage. It was considered that a S-CVI/Ave ≥ 0.9 and S-CVI/UA ≥ 0.8 meant excellent content validity.

The health care experts were asked to suggest changes that would improve language understandability or EQI content relevance whenever they assigned a score lower than 3 to an indicator. For an indicator to be accepted and become part of the EQI instrument, at a first stage, it was required that at least two of the three experts gave it a score of 3 or 4. In case of a rejection, a detailed analysis of the indicator was performed and, after considering the experts’ feedback, a second round took place to see if consensus concerning its relevance, understandability and meaning was reached. In that case, the indicator was included; otherwise, it was definitely excluded. At the end of the process, the definitive list of EQI in Portuguese was obtained.

Next, expert P5 translated the 139 QI from the ACOVE-3 checklist back into English. The applied languages experts (P6, P7) were asked to assess these QI (in English) concerning their semantics, using a 4-point concordance scale: 1 – Strongly disagree, 2—Disagree, 3—Agree, 4 – Strongly agree to state if they agreed that the retranslated version corresponded to the original one. Whenever the concordance score given to a QI was 1 or 2, the expert was asked to explain why. Experts were also asked to report issues related to the similarity between the versions.

### EQI validity, acceptance and usefulness assessment

The validity of the EQI instrument—i.e., its ability to measure what it is supposed to measure [37] was assessed in terms of its face validity (ability to be understandable and relevant for the targeted population [36, 38]), content validity (ability to reflect the domain of interest and the conceptual definition of the involved constructs [36, 37, 39]), and responsiveness (ability to detect change when a patient health status improves or deteriorates [36, 37]). This validation was performed during and after the cross-cultural adaptation (phases 2 and 3, Table 1).

Health care experts P2, P3, and P4 analyzed the understandability (syntax and semantics), the conceptual validity and relevance of the Portuguese version of the EQI (face and content validity). Applied languages experts P6, and P7 analyzed the semantic equivalence (detection of translation errors related with meaning) of the of the retranslated ACOVE-3 (in English) by comparing it to the original version of the instrument.

The focus groups were used to gain a deeper understanding of the fundamentals, processes and contexts that shape patients' interactions with health care professionals [40]. These meetings were hosted to elicit and analyze past experiences and views of the health care professionals in order to: a) discuss, identify, and validate EI that uncover inequities in the access of vulnerable older people to health care; b) determine a comprehensive list of standard terms to register inequities during health care consultations; and c) obtain health care professionals’ perceptions about the usefulness of the EQI checklist.

The focus groups qualitative content analysis comprised the phases of preparation, organization, and reporting [41]. Preparation involved defining the samples; deciding on the type of content analysis—directed for FG1 and FG2, and conventional for FG3 and FG4 [42]; preparing the discussion guides; and deciding how to collect data and the unit of analysis. The fifteen participants in the four focus groups (Additional file 1) were selected through purposeful sampling aimed at gathering PHC professionals that provided care to vulnerable older people. All the participants were from Minho, a region in the North of Portugal. The discussion guide of FG2 was composed of targeted questions, the guides for FG1, FG3, and FG4 of open-ended questions.

The discussions lasted around one hour, occurred in the PHC center, and were audio-recorded with the formal consent of all participants; records were then transcribed. Each meeting started with the moderator presenting the purpose of the focus group. Following, the participants interacted by departing from previous opinions, which resulted in a gradual co-construction of a consensual narrative. The topics of discussion were gradually introduced by the moderator that recalled to prior findings as needed. The moderator also intervened to: a) refocus the discussion on the theme; and b) reinforce and synthetize the contributions of the participants. Participants’ views were grounded in the context of everyday situations and reflected their lived experiences.

FG1 discussed the importance of EI complementary to QI. The discussion evolved around the equity concept, the dimensions considered to identify situations of inequity, and eventual difficulties in the application of the EI. FG2 started with the presentation of a list of descriptors to be used in the context of a consultation to identify situations of inequity in every equity dimension (derived from findings of the literature review). Next, there was a debate to confirm (refute) each descriptor and complete the list. Data collected in these two focus groups was explored through directed content analysis (that uses prior research to identify key concepts or variables as initial coding categories) [42] because equity is a topic that can be prone to biased or emotional opinions. The categories used for the equity concept, the dimensions of access equity, and the implications for health resulting from key variables were obtained in recent studies [5, 25, 43]

Looking at ACOVE-3 as the reference, FG3 and FG4 discussed the following topics: what are the clinical conditions of vulnerability; and what are the limitations of the QI used in older people health care management at the time of the study (PNSPI^1^); are the EQI understandable and relevant; what are the (dis)advantages of the proposed EQI; and what will be the difficulties of applying the instrument in practice. To analyze the information generated in these focus groups, since the participants were experienced in quality indicators, conventional content analysis (that lets categories flow from the data) [42] was used so that a wide variety of opinions around the discussed themes was obtained. These two focus groups also discussed EQI responsiveness and acceptance (i.e., the extent to which people delivering or receiving a health care intervention consider it to be appropriate, based on anticipated or experienced cognitive and emotional responses to the intervention [44]).

In the organization phase, a set of rules for coding was developed (e.g., for the *Equity* category, all phrases related with defining or difficulties in defining equity should be coded under the subcategory *concept*). The unit of analysis was the record transcription. Given the low abstraction levels required to: 1) link raw data to predetermined categories, in the case of FG1 and FG2, or 2) group and categorize phrases according to their meanings, similarities and differences, in the case of FG3 and FG4, these processes were relatively simple. The discussion of the equity concept was the exception. In the analysis of transcripts of FG1 and FG2, doubtful categorizations were discussed among the research team. In the case of FG3 and FG4, categories emerged well differentiated from the transcripts and situations of doubt were rare. Coding was double checked.

To explore the usefulness of the EQI—i.e., the ability of the instrument to help the health professional(s) during the consultation, a set of 30 past clinical records was analyzed. Clinical records that fulfilled the following inclusion criteria were randomly selected: a) age of patient > 65 years, b) vulnerable patient (at risk of functional decline or death in two years) according to the frailty criteria of [45], and c) regular and current user of the PHC center. Around 60% of the selected patients were women; patients were, on average, 73 years old; around 93% did not comply with active life (good nutrition, exercise) recommendations; only around 7% had low risk of falls, 80% understood medication prescriptions (doses and purpose); around 17% consumed alcohol occasionally, one third of the men were alcohol dependent. The clinical record of each patient was analyzed back until when the patient completed 65 years relatively to each EQI, using a verification table built for the purpose. The analysis was made by the first author, who holds a Bachelor of Science in Nursing, a Master of Science in Management and has worked in PHC for several years, and involved looking for registries concerning the sequential follow-up of occurrences as recommended in the EQI and their dichotomous classification as *registered* or *not registered* (e.g., if at given moment a patient was diagnosed with a medical condition that needed subsequent actions, and the medical record did not contain information about such actions in the appropriate period of time, the EQI at stake was considered as *non-registered*). The comparison between real past health care actions and the desirable actions guided by the EQI provided some hints about the potential health gains that could have been obtained by using the EQI, i.e., the usefulness of the instrument.

## Results

### Literature review

Knowledge about inequities in health care use of specific groups remains scarce [46]. Following the database search, after duplicates were removed, the abstracts of 118 retrieved studies were read and their full text was skimmed. Four papers were added to the retrieved studies due to their relevance for the study (these documents were identified in the references’ list of read articles). Nineteen studies met the inclusion criteria. A flow-chart of the study selection process is available in Additional file 2.

In Table 2, a summary of the 19 reviewed studies is presented. Most of the studies have been directed at only one dimension of equity and considered a limited number of determinants of health (in)equity. Wong et al. [64] was an exception: the study developed a set of EI for the Canadian PHC services considering four dimensions (Inequity-responsive care; Culturally-safe care; Trauma/violence-informed care; and Contextually-tailored care) and 17 indicators.

**Table 2:** Equity literature review

Equity dimensions	Study	Related to		Determinants or indicators of health (in)equity
		**Primary health care**	**Older people**	
Socioeconomic positions	[46]	x		Gender
				Socioeconomic position
				Education
Health care needs				Self-rated health
				Physical limitation or illness after injury
				Chronic diseases
				Mental Health
	[47]		x	Age
Access to primary health care centers	[48]	x		Official residency
	[49]	x	x	Physical accessibility
	[50]		x	Racial and ethnic differences
	[51]	x		Marginalized groups (e.g., homeless, LGBT)
	[52]	x		
	[53]	x		
	[54]		x	Spatial distance
Financial barriers	[55]	x		Health cost
	[56]	x		Disability pension (reimbursement system)
	[57]		x	Health insurance coverage
	[58]		x	Inappropriate drug treatment
Socio-economic barriers	[59]		x	Pension support
				Low social caste
Social barriers	[60]		x	Age, sex, country of birth, place of residence
	[61]		x	Ageism
	[62]			
	[63]		x	Social isolation and loneliness
Culturally-safe care, Inequity-responsive care, Trauma/violence-informed care, Contextually-tailored care	[64]	x		Provide ongoing training for all staff Staff work to their full scope of practice Health equity in Vision and Mission Statements Strategies to support staff to deal with the care Emotional impact of work Staff demonstrate culturally safe care Patients’ level of trust in staff Interprofessional collaboration Coordinate with community services Collaborate with other health departments Create processes to identify and follow-up patients Tailor services Examine how staff members’ verbal and non-verbal interactions impact patients Develop mechanisms to integrate input from all staff members Assess levels of improvements in patients’ quality of life Increased knowledge and skills Track patient-population unmet health care needs Assess patients’ levels of confidence in managing their health

Based on the determinants or indicators of health (in)equity identified in the literature (Table 2) and on Equity-Oriented Health Care (EOHC) orientations [43], a list of equity standard descriptors for PHC of older adults organized into domains was discussed in FG2 and, as a result, 27 standard descriptors for the 5 equity dimensions (Table 3) were obtained.

**Table 3:** Operational definitions of the five equity dimensions

Equity Dimensions	Standard descriptors
Availability	- unavailability of the drug (at the pharmacy / hospital)
	- unavailability of agenda for consultation/exam appointment
	- opening hours of the health care institution are not convenient
	- unavailability of specialized consultation / examination / physical therapy in due time
	- unavailability of specialized medical transport (ambulance to transport patients for treatment)
	- illiteracy regarding the services provided by the health institution
	- other (please describe)
Accessibility	- inexistence of primary health care centers / laboratories / physical therapy clinics
	- long distance to the assigned health care / physical therapy site
	- inexistence of public transportation to the assigned health care / physical therapy site
	- the health care center does not prioritize services that specifically address the local population’s demographics and needs
	- patient does not have openness to talk about sensitive issues such as mental health problems, substance use, and experiences of violence
	- other (please describe)
Affordability	- high costs of transportation to the assigned health care / physical therapy site
	- high cost of the medication / treatments prescribed
	- high cost of the exam prescribed
	- high cost of the consultation (user fees)
	- other (please describe)
Quality	- long waiting time at the consultation / examination site
	- long time between the appointment and the consultation / examination
	- unfriendliness of the health care professional / administrative team
	- bad physical conditions of the health care site
	- scheduling error
	- unsatisfactory previous experience
	- other (please describe)
Acceptability	- lack of trust in the health professional
	- the doctor did not refer for the examination, consultation of specialty or other care service the patient expected
	- beliefs/myths of the patient that are contrary to medical science
	- lack of spaces for interactions that are physically, emotionally, and culturally safe in the health care site
	- the patient had not understood the purpose of the prescribed treatment
	- in the absence of observable clinical findings, the concerns of the patient were not valued
	- other (please describe)

Since no comprehensive set of EI that could be incorporated into the ACOVE-3 structure was identified, the five equity dimensions considered to develop the EI (Additional file 3)—*Availability*, *Accessibility*, Cost (*Affordability*), *Quality*, and *Acceptability*—were based on the work of Furtado and Pereira [65] and Obrist et al. [66]. *Acceptability* assesses whether the provision of health care suits the users' needs and expectations [65, 66]. *Quality* refers to the patient’s or caregiver’s judgment about the quality of care, which may possibly be influenced by past experiences or the reputation of the health care organization, being more related to the user’s perceptions about adequacy, safety, trust, outcomes, and experience (that is, user satisfaction) than to the technical aspects of health care quality (e.g., diagnosis accuracy) [65, 66]. *Affordability* (costs) considers if the prices of services for the users fit their income and ability to pay and includes directs cost of purchasing the care, such as the non-reimbursed part of a drug, but also the cost of transport to the health care site or costs of waiting for care appointments [65, 66]. *Availability* is the existence of an adequate portfolio of health care services [65, 66].

### EQI validity and acceptance assessment

The final EQI instrument is composed by QI for twelve clinical conditions and EI for the five equity dimensions aimed at ten of the clinical conditions (in the EQI assessment phase – FG3, it was determined that, for *urinary incontinence* and *screening and prevention*, no EI should be included because the intention was to monitor access equity and all care related to these conditions would take place at the PHC center not involving referrals). Additional file 3 shows the complete set of EI; the definitive version of the instrument, in Portuguese, is available here^2^. An example of an EI is:

*“IF pharmacological therapy is prescribed to the Vulnerable Elder and the patient has not started it, THEN the health professional must choose the cause from the following:*

- *unavailability of the drug (specify the reason);*
- *geographical /physical inaccessibility;*
- *unaffordable costs to be incurred (specify which);*
- *lack of quality associated with the process (professional service, schedules);*
- *refusal of the user /caregiver (therapy does not correspond to her/his expectations)”.*

In Table 3, a list of suggested standard descriptors for most usual inequity situations is provided. These descriptors were derived from the EOHC [43] and validated and completed by FG2.

Relatively to the EQI, the analysis by the health care experts (P2, P3, P4) led to a total consensus in terms of face validity (score average of 4). In terms of content validity of the QI, there was an initial rejection of 12% of the QI by expert P2 (score average of 3.61), 1% by P3 (score average of 3.96) and 4% by P4 (score average of 3.91). Concerning the content validity of the EI, there was an initial rejection of 11% of the indicators by P2 (score average of 3.57), and of 5% by P4 (score average of 3,89) occurs. P3 did not reject any indicator (score average of 4). Following the suggestions of these experts, the text of the problematic EQI was changed and, in a new round, only 2% of the indicators were rejected: 4 QI of the clinical condition Depression, and 2 EI related with Medication Use. The results relative to the EI were S-CVI/Ave of 0.89 for P2, 1.00 for P3, and 0.95 for P4. In relation to the QI, the S-CVI/Ave was 0.88 for P2, 0.99 for P3, and 0.96 for P4. In terms of S-CVI/UA, the results were 0.83 (23 of 139 indicators were rejected) for QI, and 0.84 (5 of 31 indicators were rejected) for EI. Twenty-three out of 139 QI were rejected (20 due to item CVI of 0.67, and 3 with item CVI of 0.33); the same happened to 5 out of 31 EI (4 with item CVI of 0.67 and 1 with item CVI 0.33).

When the language experts (P6, P7) analyzed the QI concerning possible meaning variability in the back-translated version, all QI were accepted without need for further adjustments (score average = 4).

**Fig. 1:**
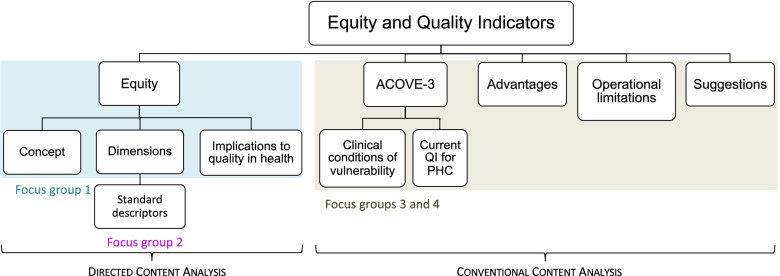
Categories derived through focus group data content analysis

The categories used for or derived from the qualitative data analysis of the information collected through focus groups are depicted in Fig. 1.

In the *Equity* category, discussed in FG1, the following points were evident:

*Concept*: There was no comprehensive and consensual notion of equity; therefore, the participants concluded that an effort should be made to communicate to health care professionals what equity in health care access means.

*Dimensions*: Only three out of the five *dimensions* of equity in health care access were mentioned, namely: *accessibility*, *affordability* and *acceptability*, further emphasizing the need for an instrument like the one developed in this study. E.g., a participant stated “*(…) the indicators we currently have expose the undervaluation of equity in health care access”* (P12);

*Implications to quality in health*: The EI that were available at the moment of the data collection were generic—“*(…) the current indicators to evaluate access are general, for everyone; the older people, who are a vulnerable group, as well as children or pregnant women, should be given more attention.”* (P11); the suggested EI are much better directed at older people.

According to the participants, the monitoring of health care equity (currently undervalued or fragmented) is directly proportional to the *quality* of the service and the satisfaction of the users:

“*If there is no equity, there is no quality, they compromise each other.”* (P8).

“*If we monitor the equity levels that the population faces, we will also have better levels of health and population satisfaction, essentially, best quality of care”* (P12).

In the *ACOVE-3* category, the following points emerged (from FG3 and FG4):

The clinical conditions of vulnerability considered in the PNSPI were described as generic (i.e., only related with the physiological decline associated with aging, and processes of co-morbidity) and it was stated that the proposed EQI checklist “*(…) addresses several aspects still undervalued by health professionals.”* (P10). A consensus about the understandability and relevance of the Portuguese version of the QI emerged, and no one suggested changes to the checklist.

The quality indicators *directed at older people implemented in PHC* at the moment of the meetings were limitative (only four indicators for the purpose were identified), did not raise awareness or provided guidance to the health care teams about the need for an integral assessment of the older person: “*(…) it’s only when we see the* [proposed] *checklist that we become aware that there are many conditions that we do not value, because we focus on very specific complaints from the older adults”* (P22). In terms of clinical governance, the participants emphasized the importance of obtaining lessons regarding the quality of the care processes that, in the future, can result in an improvement of health care service. At this level, the participants highlighted the need for more suitable performance metrics.

Relatively to the *Advantages*, the participants considered that, once integrated in the work activities of the health care teams, the proposed EQI instrument would fulfil the previously identified needs by:

- considering a set of clinical conditions specifically directed at the vulnerable older adults: “*(…)* [the proposed checklist] *presents a complete and adequate notion of the most prevalent needs of the older patients”* (P18);
- fitting into the Portuguese PHC;
- promoting health, preventing/ screening disease, and supporting disease management: “*(…) through the application of ACOVE-3, a group of specific interventions that, if implemented in the clinical practice, can result in health gains, emerges (…).* (P14)”; there was consensus about the responsiveness of the EQI checklist.
- favoring the obtention of information about the quality of health care, the adoption of good practices, and the occurrence of inequity situations.

Concerning the *Operational limitations*, the most problematic issue pointed out by the participants was the length of the instrument that, given the constrains imposed by tight consultation times, can create some resistance to implementation: “*(…) as* [the proposed EQI checklist] *is extensive, there will be resistance from the health professionals.”* (P13); “*(…) something that takes up time in the consultation is something that the health professionals always try to avoid (…)”* (P19); *“Since* [the proposed EQI checklist] *requires a possible change in practice, there are professionals who will probably not adhere.”* (P22).

In terms of *Suggestions*, the participants highlighted that the advantages of the EQI should be communicated to health professionals in order to motivate effective use and the integration of the EQI in the information systems used by the teams. They also suggested that a pilot implementation of a reduced version of the instrument in a PHC center was conducted in order to show the advantages of the EQI implementation.

In summary, given the near-absence of specific indicators for vulnerable older adults, the health professionals expressed positive perceptions concerning the intention to use the developed EQI.

### EQI usefulness

The usefulness of the EQI instrument was further assessed through the analysis of 30 patient clinical records. Table 4 contains the percentage of classifications as *non-registered* EQI that resulted from that analysis (i.e., situations when a quality or equity issue should have been registered and it was not). It was found that a significant part of the issues addressed by the proposed EQI had not been registered by the health professionals that interacted with the older patients (on average, 76.6% of the QI-related situations that should have been registered, and 96.7% of the EI-related situations that should have been registered were not written in the clinical records), which was expectable because the EQI were not implemented at the time of the health care provision analyzed, but highlights that some important quality or equity questions may had been overlooked. From the 24.4% of QI-related situations that had been registered in the clinical records, 55% concerned problematic situations (i.e., quality problems) that could have been avoided if the EQI were already at place. In the case of the EI, the registrations were rare.

**Table 4:** Data from 30 patient clinical records

Clinical condition (from ACOVE-3)	EQI Checklist			Past clinical records	
	**# of EQI**	**# of QI (# of situations to check)**	**# of EI**	**% of non-registered situations (record** $\times$ **indicator)**	
				**QI**	**EI**
Continuity and Coordination of Care	12	8 (12) ^(a)^	4	75.0%	100.0%
Dementia	19	16 (20)	3	90.0%	100.0%
Depression	29	18 (30)	11	93.6%	99.7% ^(b)^
Falls and Mobility Problems	14	12 (12)	2	81.1%	85.0%
Hearing Loss	9	7 (7)	2	71.0%	100.0%
Medication Use	27	24 (24)	3	73.8%	95.6%
Pressure Ulcers	12	10 (14)	2	80.2%	93.3%
Screening and Prevention	14	14 (14)	0	39.0%	-
Sleep Disorders	12	10 (10)	2	69.7%	100.0%
Malnutrition	7	6 (17)	1	88.6%	93.3%
Urinary Incontinence	9	9 (9)	0	77.8%	-
Vision	6	5 (5)	1	80.0%	100.0%
**Total**	**170**	**139 (205)**	**31**	**76.6%**	**96.7%**

(a) 8 (12) in *# of QI checked* means 8 indicators and 12 situations to be checked (E.g., Quality Indicator #2—Medication Follow-up in the Outpatient Setting of Continuity and Coordination of Care requires the verification of 3 situations.); (b) Of the 30 clinical records analysed, only had information about only 1 indicator: % of non-registered situations = 99.7% = $1-\left(\frac{1\;record\times1\;indicator}{30\;records\times11\;indicators}\right)\times100$

## Discussion

The EQI proposed are a structured and complete set to track health care quality and equity conditions that are relevant for vulnerable older adults, thus helping health professionals to provide a better-quality service, for example, by avoiding misdiagnosis that, when this population is involved, can result in severe health consequences. The various phases of the design played different roles in development of the EQI: the EI were identified through a scoping review; a cross-cultural adaptation of ACOVE-3 provided the appropriate structure to incorporate the EI; and the focus groups and the retrospective analysis of patient clinical records enabled an overall appraisal of the benefits and acceptance of the EQI checklist.

This work contributes to the effort of improving quality indicators in PHC through the transfer of ACOVE from the USA to other countries: other researchers have performed cross-cultural adaptations of this instrument to The Netherlands [67]—8 conditions and 72 indicators adapted); Switzerland [68]—56 indicators; and the UK [69]—16 conditions and 102 indicators. As in these studies, we have not adapted all clinical conditions and indicators of the original ACOVE instrument, and differences occur due to the specific needs of each country's PHC system.

The categorization of equity in *Availability*, *Accessibility*, Cost (*Affordability*), *Quality*, and *Acceptability* [65, 66] is not unanimous but includes the most common equity concerns. For example, Goddard and Smith [70] studied inequities in access to some types of health care (such as general practitioner consultations or preventive medicine) in the UK, considering four dimensions: availability, quality, costs and information. The information dimension is somehow related with *accessibility* and *acceptability* because the availability of certain services is not equally known by all population groups. *Accessibility* includes the physical or geographical accessibility of care [65, 66], a common dimension in equity studies [49, 54].

The choice of standard descriptors for the EI followed the orientations of EOHC [43] that aims to mitigate the power imbalances, systemic barriers, and dismissive attitudes that people living in marginalizing conditions often encounter when interacting with the health care sector. Providing care tailored to patients’ social circumstances may help create a sense of mutual respect and trust necessary for the patient to engage with health care over time [71]. From a practical perspective, the report of inequity situations should be as standardized as possible in order to facilitate description and subsequent analysis: if a count of a standard reported situation is high, decision makers can rapidly identify the problem and act to solve it; if, on the other hand, each health professional can write a non-standard description, the analysis will probably be difficult, eventually leading to inconclusive results and lack of action.

The few health equity studies that have addressed older people typically used non-systematic surveys [72] or generic data from NHS information systems [47] to analyze the equity status (e.g., by comparing admissions of older adults with those of other population groups). There has been a gap concerning the systematic collection of dedicated information that can support health care and policy decision making and ultimately contribute to improve care provided to older adults. This study was directed at favoring the collection of such information.

The acceptance of the EQI instrument by health care professionals seems evident. However, it would be essential to incorporate it into existing information systems – e.g., electronic medical record systems, as it was done for ACOVE-3 in the Netherlands [73], and to explain the advantages of its use to primary health care general practitioners and nurses. Also, the application of the proposed list of standard terms to describe the causes of situations of inequity can expedite the use of the instrument.

It would be interesting to further explore the automatic evaluation of at least some of the EQI, thus answering the concerns of health care professionals about the length of the instrument given the normal time of a consultation. The analysis of a sample of patient clinical records pointed towards the usefulness of the instrument. Also, starting to use the instrument to collect and analyze data about the quality and equity of the primary health care to vulnerable older adults and disclose the results of that assessment will contribute to the acceptance of the instrument and spread it as a benchmark tool, hopefully, improving health care.

The use of the proposed EQI instrument will likely improve the health care service provided to a fragile population in Portuguese speaking countries and communities. The developed EI can be incorporated into ACOVE-3 based instruments also in other countries, improving the screening of inequity in the access to health care services by vulnerable older adults. It must be noted that, in the future, the proposed EI should be further developed by adding inequity mitigation actions to be taken when an occurrence of inequity is detected. These actions should be determined after EI implementation and some period of information gathering, and must be contextually adapted.

In Portugal, there were no systematized EI for the vulnerable older adults. The incorporation of the EI in the structured ACOVE checklist favors the registration of detailed information by health professionals. That detail enhances data analysis and contributes to more accurate information about the reality. In practice, by filling the form about one of the five equity dimensions, health professionals are invited to register why some clinical conditions were not satisfied. In each one of them, they also have the possibility to detail the causes of the observed inequity.

Because of the Covid-19 pandemic situation, the main limitation of this study was the overwork of the health care professionals that limited their availability to participate in the research. A wider participation would have strengthened the validation of the study. As the clinical records can only be consulted by health professionals, it was not possible to double check the analysis of these data.

## Conclusions

Health organizations and authorities will have a source of information that can support decision making if data concerning situations of lack of quality and inequity in primary health care of older adults start to be systematically collected and analyzed. Contributing to that effort, this study developed equity indicators for ACOVE-3, and undertook the first cross-cultural adaptation and validation of ACOVE-3 into Portuguese. The EQI instrument was validated and assessed by general practitioners and nurses through expert opinion and focus groups, and was well accepted by these health care professionals.

The developed equity and adapted quality indicators were tailored to PHC in the Portuguese national health system, but they can easily be adapted to secondary care or to the health care systems of other countries.

## Supplementary Information

**Additional file 1.  **Experts and focus groups participants involvement.

**Additional file 2. **Literature review flow chart.

**Additional file 3. **List of the equity indicators.

## Data Availability

The expert opinion and focus groups data collected and analysed during the current study are available from the corresponding author on reasonable request. The clinical records data that support some findings of this study are available from ARS Norte but restrictions apply to the availability of these data, which were used under license for the current study, and so are not publicly available.

## Abbreviations

ACOVE: Assessing Care of Vulnerable Elders
WHO: World Health Organization
QI: Quality indicators
PHC: Primary health care
EQI: Equity and quality indicators
EOHC: Equity-Oriented Health Care

## Footnotes

1 Plano Nacional de Saúde da Pessoa Idosa (Older People National Health Plan).

2 doi: 10.17632/4h9637mt8y.1
