# Supplementary material for: Assessing equity and quality indicators for older people – Adaptation and validation of the Assessing Care of Vulnerable Elders (ACOVE) checklist for the Portuguese care context
Source: BMC Geriatr. 2022 Jul 6;22:561. doi: 10.1186/s12877-022-03104-5 (PMC9256534; doi:10.1186/s12877-022-03104-5)
Supplement: Supplementary file 1 — Additional file 1. Experts and focus groups participants involvement. [file 12877_2022_3104_MOESM1_ESM.docx]

**Additional file 1**

Experts and focus groups participants involvement

| **Description** | **Participants** | **Role** | **Qualifications** |
| --- | --- | --- | --- |
| Expert in translation and communication | Expert 1: P1 | - Translate Assessing Care of Vulnerable Elders (ACOVE)-3 checklist from English into Portuguese | - Master in Translation and Multilingual Communication - Member of BabeliUM – Language Center of the School of Arts and Humanities School, University of Minho, Portugal - Specialized in the translation of data collection instruments in the clinical area. - Portuguese Native speaker |
| Expert panel – Health care | Expert 2: P2 | - Analyze the understandability (syntax and semantics) of the language used, and the conceptual validity and relevance of the equity and quality indicators (EQI) for the older adults - Face validity and content validity;   - Prepare a critical summary. | - PhD in Medicine; - Professor at a Medicine School in Portugal; - Author of research in health communication, scale development and validation, and health literacy published in international journals |
|  | Expert 3: P3 |  | - PhD in Nursing - Professor at a Nursing School in Portugal - Author of research about health care of older people, and scale development published in international journals - Older adult |
|  | Expert 4: P4 |  | - Master in Medicine with specialization in General and Family Medicine - General Practitioner at a primary health care center in the north of Portugal - Years of experience in health care of vulnerable older adults - Older adult |
| Expert in translation and communication | Expert 5: P5 | - Retranslate ACOVE-3 checklist to English. | - BSc in Education - Native from an English-speaking country - Resident in Portugal for more than 10 years. |
| Expert panel - Applied Languages | Expert 6: P6 | - Evaluate the semantic equivalence (detection of translation errors related with meaning) of the retranslated ACOVE-3 to English by comparing the original version with the retranslated one; - Prepare a critical summary. | - Master in Translation and Multilingual Communication - Members of BabeliUM – Language Center of the School of Arts and Humanities School, University of Minho, Portugal, - Years of experience in linguistic support to health and health care studies |
|  | Expert 7: P7 |  |  |
| Focus groups - Health Professionals | Focus Group 1:  P8, P9, P10, P11, P12 | - Discuss the equity concept and the dimensions to be considered to identify iniquitous situations; - Identify equity indicators (EI) to assess inequities in vulnerable older people primary health care access; - Discuss eventual difficulties of applying the EI. | - General Practitioners (P11, P12), and Nurses (P8, P9, 10) - Years of experience in providing health care to vulnerable older adults |
|  | Focus Group 2:  P8, P9, P10, P11, P12 | - Discuss the standard descriptors to register inequities in vulnerable older people primary health care access. | - General Practitioners (P11, P12), and Nurses (P8, P9, 10) - Years of experience in providing health care to vulnerable older adults |
|  | Focus Group 3:  P13, P14, P15, P16, P17 | - Analyze the understandability (syntax and semantics) of the language used and the relevance (content) of the EQI - Face and content validity; - Discuss EQI responsiveness, acceptance, and usefulness; - Discuss eventual difficulties of applying the EI. | - General Practitioners (P15, P16, P17), and Nurses (P13, P14) - Years of experience in providing health care to vulnerable older adults |
|  | Focus Group 4: P18, P19, P20, P21, P22 |  | - General Practitioner (P22), and Nurses (P18, P19, P20, P21) - Years of experience in providing health care to vulnerable older adults |
