# Supplementary material for: Assessing equity and quality indicators for older people – Adaptation and validation of the Assessing Care of Vulnerable Elders (ACOVE) checklist for the Portuguese care context
Source: BMC Geriatr. 2022 Jul 6;22:561. doi: 10.1186/s12877-022-03104-5 (PMC9256534; doi:10.1186/s12877-022-03104-5)
Supplement: Supplementary file 2 — Additional file 2. Literature review flow chart. [file 12877_2022_3104_MOESM2_ESM.docx]

**Additional file 2**

Literature review flow chart
