# Supplementary material for: Assessing equity and quality indicators for older people – Adaptation and validation of the Assessing Care of Vulnerable Elders (ACOVE) checklist for the Portuguese care context
Source: BMC Geriatr. 2022 Jul 6;22:561. doi: 10.1186/s12877-022-03104-5 (PMC9256534; doi:10.1186/s12877-022-03104-5)
Supplement: Supplementary file 3 — Additional file 3. List of the equity indicators. [file 12877_2022_3104_MOESM3_ESM.docx]

**Additional file 3**

List of the equity indicators

| **Clinical condition** | | **Equity Indicator** | **Inequity causes** | | | | |
| --- | --- | --- | --- | --- | --- | --- | --- |
|  |  |  | Unavailability | Inaccessibility | Unaffordability | Lack of quality | Unacceptability/ Unacceptance |
| **Continuity and Coordination of Care** | | Medication follow-up |  |  |  |  |  |
|  |  | ***IF*** *a vulnerable elder (VE) was prescribed a new medication and he or she has not started it,* ***THEN*** *inquire and register the causes on the patient medical record and in a database of inequity occurrences.* | * | * | * | * | * |
|  |  | Specialist consultation follow up |  |  |  |  |  |
|  |  | ***IF*** *a VE was referred to a specialist consultant physician and the consultation did not take place within six weeks of referral,* ***THEN*** *inquire and register the causes on the patient medical record and in a database of inequity occurrences.* | * | * | * | * | * |
|  |  | Follow-up after Hospital Discharge |  |  |  |  |  |
|  |  | ***IF*** *a VE was discharged from a hospital to his or her home and a follow-up visit or documented telephone contact did not take place within six weeks of discharge,* ***THEN*** *inquire and register the causes on the patient medical record and in a database of inequity occurrences.* | * | * | * | * | * |
|  |  | Medical Visits and Appointments after Hospitalization |  |  |  |  |  |
|  |  | ***IF*** *a VE was discharged from a hospital to his or her home or to a nursing home, and the hospital medical record specifies a follow-up appointment for a physician visit or to a treatment that did not take place within six weeks of discharge,* ***THEN*** *inquire and register the causes on the patient medical record and in a database of inequity occurrences.* | * | * | * | * | * |
| **Dementia** | | Laboratory testing |  |  |  |  |  |
|  |  | ***IF*** *a VE with newly diagnosed dementia did not take laboratory tests,* ***THEN*** *inquire and register the causes on the patient medical record and in a database of inequity occurrences.* | * | * | * | * | * |
|  |  | Screening for depression |  |  |  |  |  |
|  |  | ***IF*** *a VE with dementia was not screened for depression during the initial evaluation,* ***THEN*** *inquire and register the causes on the patient medical record and in an inequity occurrences database.* | * | * | * | * | * |
|  |  | Stroke prophylaxis |  |  |  |  |  |
|  |  | ***IF*** *a VE with dementia prescribed with the appropriate prophylaxis did not start it,* ***THEN*** *inquire and register the causes on the patient medical record and in a database of inequity occurrences.* | * | * | * | * | * |
| **Depression** | | Recognizing Depression |  |  |  |  |  |
|  |  | ***IF*** *a VE presents depression symptoms and was not referred to a mental health professional or, if referred, the consultation did not take place within two months of referral,* ***THEN*** *inquire and register the causes on the patient medical record and in a database of inequity occurrences.* | * | * | * | * | * |
|  |  | Suicidal Ideation |  |  |  |  |  |
|  |  | ***IF*** *a VE with thoughts of suicide was referred for psychiatric evaluation or to psychiatric hospitalization that did not take place,* ***THEN*** *inquire and register the causes on the patient medical record and in a database of inequity occurrences.* | * | * | * | * | * |
|  |  | Depression treatment |  |  |  |  |  |
|  |  | ***IF*** *a VE was prescribed with antidepressant treatment, psychotherapy, or electroconvulsive therapy (ECT) that has not started within 2 weeks of prescription,* ***THEN*** *inquire and register the causes on the patient medical record and in a database of inequity occurrences.* | * | * | * | * | * |
|  |  | Psychiatric Referral for Psychotic Depression |  |  |  |  |  |
|  |  | ***IF*** *a VE presents depression with psychotic features and was referred to psychiatric consultation or was prescribed with new treatment that has not taken place within a week of referral or prescription,* ***THEN*** *inquire and register the causes on the patient medical record and in a database of inequity occurrences.* | * | * | * | * | * |
|  |  | Electrocardiogram for tricyclic use |  |  |  |  |  |
|  |  | ***IF*** *a VE with a history of cardiac disease started on a tricyclic antidepressant, and a baseline electrocardiogram (ECG) was not be prescribed or did take place,* ***THEN*** *inquire and register the causes on the patient medical record and in a database of inequity occurrences.* | * | * | * | * | * |
|  |  | Monitoring depression |  |  |  |  |  |
|  |  | ***IF*** *a VE was prescribed with an antidepressant treatment that was not initiated,* ***THEN*** *inquire and register the causes on the patient medical record and in a database of inequity occurrences.* | * | * | * | * | * |
|  |  | ***IF*** *a VE is being treated for depression and, at each treatment visit, suicide risk has not been evaluated,* ***THEN*** *inquire and register the causes on the patient medical record and in a database of inequity occurrences.* | * | * | * | * | * |
|  |  | ***IF*** *a VE has not shown any significant response to symptoms after 6 weeks or responds only partially after 12 weeks of treatment and a follow-up consultation or documented telephone contact did not take place,* ***THEN*** *inquire and register the causes on the patient medical record and in a database of inequity occurrences.* | * | * | * | * | * |
|  |  | ***IF*** *a VE has responded only partially after 12 weeks of treatment and a switch to a different medication class, or addition of a second medication to the first (if the initial treatment includes medication), or addition of psychotherapy (if the initial treatment was medication), or introduction of medication (if the initial treatment was psychotherapy without medication), or considering ECT did not take place,* ***THEN*** *inquire and register the causes on the patient medical record and in a database of inequity occurrences.* | * | * | * | * | * |
|  |  | The First 12 Weeks of Depression Treatment  ***IF*** *a VE has responded to antidepressant medication and has not continued on the drug at the same dose for at least six months, or at least one clinician contact (office visit or phone) during that time period, did not take place,* ***THEN*** *inquire and register the causes on the patient medical record and in a database of inequity occurrences.* | * | * | * | * | * |
|  |  | Continuation of Antidepressant Therapy |  |  |  |  |  |
|  |  | ***IF*** *a VE had three or more episodes of depression and has not taken maintenance antidepressant medication for at least the past 24 months, or follow-up consultations (at least four) or frequent documented telephone contact did not take place,* ***THEN*** *inquire and register the causes on the patient medical record and in a database of inequity occurrences.* | * | * | * | * | * |
| **Falls and mobility problems** | | Balance disturbances |  |  |  |  |  |
|  |  | ***IF*** *a VE was prescribed an assistive device for excessive balance, proprioception or swaying of the body and he or she does not use it,* ***THEN*** *inquire and register the causes on the patient medical record and in a database of inequity occurrences.* | * | * | * | * | * |
|  |  | Exercise program |  |  |  |  |  |
|  |  | ***IF*** *a VE* *was prescribed a structured or supervised exercise program for problems with walking, balance, strength or endurance that has not been initiated,* ***THEN*** *inquire and register the causes on the patient medical record and in a database of inequity occurrences.* | * | * | * | * | * |
| **Hearing loss** | Formal audiologic examination |  |  |  |  |  |  |
|  | ***IF*** *a VE that failed a hearing screening examination was not prescribed a formal audiologic evaluation within 3 months of the examination,* ***THEN*** *inquire and register the causes on the patient medical record and in an inequity occurrences database.* | * | * | * | * | * |  |
|  | Conductive hearing loss |  |  |  |  |  |  |
|  | ***IF*** *a VE present deafness in the bone conduction in audiometry and an otorhinolaryngologist consultation was not prescribed or did not take place,* ***THEN*** *inquire and register the causes on the patient medical record and in a database of inequity occurrences.* | * | * | * | * | * |  |
| **Medication** | Prescription |  |  |  |  |  |  |
|  | ***IF*** *a VE was prescribed a new drug that he or she has not started,* ***THEN*** *inquire and register the causes on the patient medical record and in a database of inequity occurrences.* | * | * | * | * | * |  |
|  | Laboratory monitoring of the angiotensin-converting enzyme inhibitor |  |  |  |  |  |  |
|  | ***IF*** *a VE was prescribed the ACE inhibitor, and serum creatinine / serum potassium were not monitored within 2-4 weeks after starting therapy and thereafter at least once a year,* ***THEN*** *inquire and register the causes on the patient medical record and in a database of inequity occurrences.* | * | * | * | * | * |  |
|  | Monitoring of loop diuretic therapy |  |  |  |  |  |  |
|  | ***IF*** *a VE was prescribed a loop diuretic and the electrolytes were not checked within 2 weeks of initiation of the treatment, and at least once a year,* ***THEN*** *inquire and register the causes on the patient medical record and in a database of inequity occurrences.* | * | * | * | * | * |  |
| **Pressure ulcers** | Risk assessment |  |  |  |  |  |  |
|  | ***IF*** *a VE presents or is at risk of developing a pressure ulcer and a weekly re-evaluation for 4 weeks, or every two weeks thereafter has not taken place* ***THEN*** *inquire and register the causes on the patient medical record and in a database of inequity occurrences.* | * | * | * | * | * |  |
|  | Management of pressure ulcer |  |  |  |  |  |  |
|  | ***IF*** *a VE presents a pressure ulcer with no improvement within 2 or 4 weeks according to its evolutionary characteristics and the appropriateness of the treatment plan has not been assessed,* ***THEN*** *inquire and register the causes on the patient medical record and in a database of inequity occurrences.* | * | * | * | * | * |  |
| **Sleep disorders** | Sleep study |  |  |  |  |  |  |
|  | ***IF*** *a VE presents daytime sleepiness, apnea or loud snoring, and a sleep study has not been prescribed or have not taken place,* ***THEN*** *inquire and register the causes on the patient medical record and in a database of inequity occurrences.* | * | * | * | * | * |  |
|  | Limb movements in sleep |  |  |  |  |  |  |
|  | ***IF*** *a VE presents limb movements during sleep and frequent awakenings or excessive daytime sleepiness and a prescribed therapy or treatment, or a consultation with a specialist physician have not taken place,* ***THEN*** *inquire and register the causes on the patient medical record and in a database of inequity occurrences.* | * | * | * | * | * |  |
| **Malnutrition** | Weight loss assessment |  |  |  |  |  |  |
|  | ***IF*** *a VE had involuntary loss of > 10% body weight over one year or less or hypoalbuminemia (< 3.5 g/dL), and an evaluation for potentially relevant causes documented in the medical record have not taken place,* ***THEN*** *inquire and register the causes on the patient medical record and in a database of inequity occurrences.* | * | * | * | * | * |  |
| **Vision** | Urgent Signs and Symptoms |  |  |  |  |  |  |
|  | ***IF*** *a VE has had sudden-onset visual changes, eye pain, corneal opacity, or severe purulent discharge and a patient examination by an ophthalmologist did not take place within 72 hours,* ***THEN*** *inquire and register the causes on the patient medical record and in a database of inequity occurrences.* | * | * | * | * | * |  |

* Answer by selecting the applicable reason from those available in Table 3.
